# Anesthetic Management of Cesarean Section in the Case of a Sextuplet Pregnancy and Polycystic Ovarian Syndrome

**DOI:** 10.7759/cureus.51473

**Published:** 2024-01-01

**Authors:** Abduljaleel Ethy Ahammedunni, Nadine Borhan Mahmoud Nour, Muhammad Sharif Allah dad

**Affiliations:** 1 Department of Anaesthesia and Intensive Care, Latifa Hospital, Dubai Health, Dubai, ARE

**Keywords:** obstetric anaesthesia, polycystic ovarian syndrome, ovarian hyperstimulation, ovulation induction, sextuplets, multiple pregnancy

## Abstract

Cesarean section in a mother with a sextuplet pregnancy is challenging for an anesthesiologist. Several perioperative complications are likely because of the overdistended uterus and associated changes in the mother. We are reporting the case of a woman with a sextuplet pregnancy who came for an emergency cesarean. She also had a background history of polycystic ovarian syndrome (PCOS) and ovulation induction for conception. Early pregnancy was complicated by ovarian hyperstimulation syndrome. She required cervical cerclage in early pregnancy. The emergency cesarean was done as she went into preterm labor and six premature babies were delivered at 29 weeks of gestation. Cesarean was done under spinal anesthesia. Preeclampsia and postpartum hemorrhage complicated the perioperative period.

## Introduction

Polycystic ovarian syndrome (PCOS) is associated with hyperandrogenism, polycystic ovaries, menstrual dysfunction, and fertility problems. Gestational diabetes mellitus (GDM), hyperlipidemia, preeclampsia, and coronary artery disease are common in PCOS. Multiple pregnancies and ovarian hyperstimulation syndrome (OHSS) are likely to occur after artificial reproduction techniques (ARTs) in PCOS [1-3]. Overdistention of the uterus can result in postpartum atonic uterus and hemorrhage. Sextuplet pregnancy is rare and is frequently associated with preterm labor and premature delivery. The case of a woman with PCOS who had a sextuplet pregnancy after ovulation induction is reported. She had OHSS requiring hospital admission in early pregnancy. Subsequently, she required an emergency cesarean for preterm labor. Problems of anesthesia for cesarean in a sextuplet pregnancy are discussed. Peripartum preeclampsia and mild postpartum hemorrhage required management in the High-Dependency Unit after cesarean section.

## Case presentation

A 31-year-old obese patient with a BMI of 34.2 presented to the emergency department in preterm labor with sextuplets at 29 weeks two days gestation. She had a history of PCOS and required multiple cycles of ovulation induction to conceive, because of inadequate follicle growth. Her previous two pregnancies were the result of ovulation induction. She was admitted and kept fasting for possible emergency cesarean section. Enoxaparin injection was put on hold. Magnesium sulfate infusion was given for 24 hours.

Her pain subsided by the next day. At this time, she had lower limb edema and itching due to cholestasis and started on ursodeoxycholic acid.

**Figure 1:**
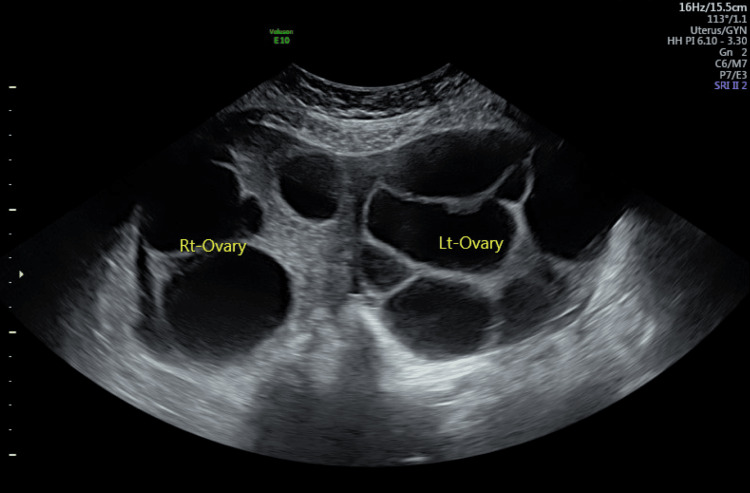
Ultrasound showing enlarged ovaries with cysts when she was admitted with ovarian hyperstimulation syndrome Rt Ovary: Right ovary, Lt Ovary: Left Ovary

**Figure 2:**
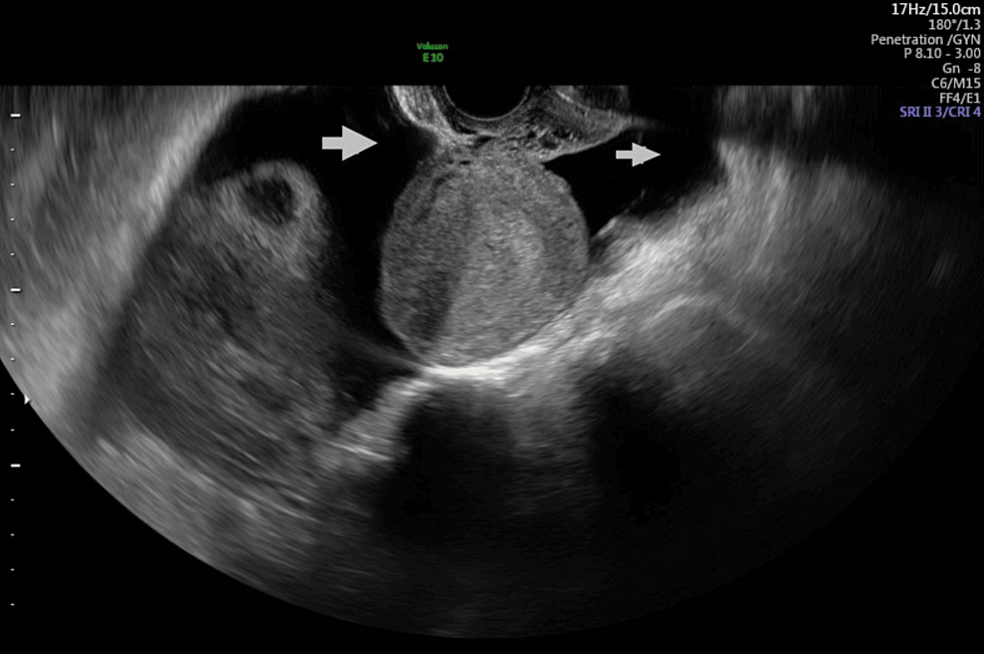
Transvaginal ultrasound during ovarian hyperstimulation Arrows show free fluid in the pouch of Douglas

**Figure 3:**
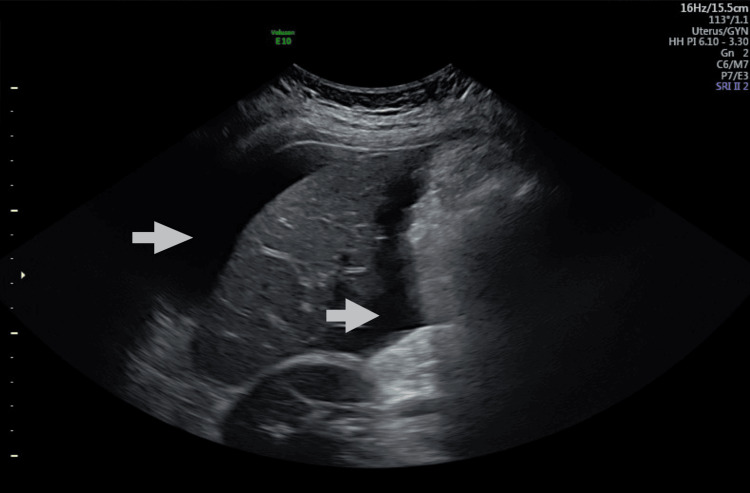
Ultrasound scan of the right upper abdomen Arrows showing fluid in the hepatorenal angle

**Figure 4:**
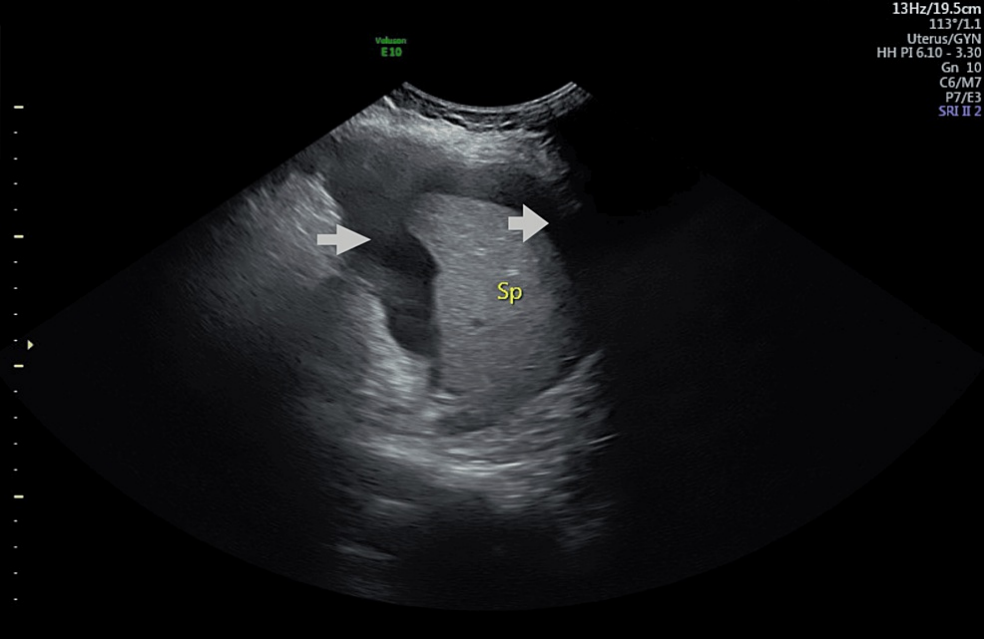
Ultrasound scan of the left upper abdomen Arrows show free fluid around the spleen. Sp: Spleen

She had prior hospital admission with abdominal pain and anorexia at five weeks of current gestation. Ultrasound examination findings were (Figure 1) enlarged ovaries with cysts sitting on the uterine fundus. The left ovary measured 11.2x11.2x10.5cm and the right measured 12.4x11.4x9.5cm. There was free fluid in the pouch of Douglas (Figure 2), hepatorenal angle (Figure 3), and around the spleen (Figure 4). There was mild bilateral pleural effusion. She was managed as OHSS in the ward with analgesia, enoxaparin, and monitoring of abdominal girth. While in hospital she developed shortness of breath and was planned for Intensive Care Unit admission but she improved subsequently. She was discharged after five days. After that, she had cervical cerclage in another hospital at 12 weeks of gestation.

She was reviewed in the outpatient department at 28 weeks' gestation, four days before the current admission. Ultrasound showed a sextuplet pregnancy. The obstetric plan was to do elective cesarean section by 34 weeks of gestation and enoxaparin, aspirin, and progesterone were advised.

Four days after admission with threatened preterm delivery, she had further pain. As she had persistent uterine contractions, she was posted for emergency cesarean with cerclage removal. She had an over-distended, tense abdomen. On vaginal examination, the cervix was 1cm, 1.5cm dilated. Necessary arrangements were made to manage the sextuplets. Neonatologists and neonatal nurses were arranged for each of the babies. Resuscitation equipment and a ventilator were arranged for each baby. Blood was arranged to manage possible hemorrhage. Blood samples were sent to the laboratory before surgery. Results are shown in Table 1. Her heart rate was 82/mt, blood pressure 157/90mmHg, and oxygen saturation on room air was 97% before the surgery.

**Table 1:** Laboratory results during hospital admission for the cesarean section. Day 5 A: before surgery, B: after surgery WBC: White Blood Cell Count; INR: International Normalized Ratio; APTT: Activated Partial Thromboplastin Time; AST: Aspartate Aminotransferase; ALP: Alkaline Phosphatase; ALT: Alanine Aminotransferase; eGFR: Estimated Glomerular Filtration Rate

Component	Reference range	Day 1	Day 2	Day 5 (cesarean)		Day 6		Day 7
				A	B	09:00am	11:00am	
WBC	3.6-11x10^9/L	4.8	3.9	5.4	8.9	7.9	7.3	6
Hemoglobin	12-15gm/dL	10.1	10.1	10.5	10.3	8.6	8.2	9.6
Hematocrit	%	31.4	31.9	32	31.6	26.5	25.3	29
Platelets	150-410x10^9/L	135	121	126	130	120	126	130
Fibrinogen	190-430mg/dL				408			
Prothrombin time	Secs			12.1	12.9			
INR	Ratio			0.92	1.0			
APTT	Secs			36.1	38.8			
Blood Glucose	60-120mg/dL			76	94			
Albumin: Creatinine Ratio	<20mg/g		101.4		211.3			
AST	0-32U/L		37		36			
Bilirubin	0-1.2 mg/dL		0.4		0.4			
ALP	35-104U/L		274		293			
ALT	0-33U/L		41		38			
Total Protein	6.6-8.7gm/dL				5.7			
Albumin	3.4-4.8gm/dL				2.7			
Serum Creatinine	0.5-0.9mg/dL		0.6		0.5			
eGFR	>60ml/min/1.73m(2)		122.3		129.9			
Sodium	136-145mmol/L		133		136			
Potassium	3.3-4.8mmol/L		3.7		4.2			
Chloride	98-108mmol/L		102		105			
Bicarbonate	20-28mmol/L		16.6		18.5			
Urea	12-40mg/dL		15		12			

Preanesthesia evaluation was done and six-hour fasting was ensured. She had borderline high blood pressure and a raised albumin:creatinine ratio. Acid aspiration prophylaxis was given and a cesarean section was done under spinal anesthesia with hyperbaric bupivacaine 11mg, fentanyl 15mcg, and preservative-free morphine 100mcg. Cesarean section was done by an obstetric consultant supported by assistants. Both ovaries were found enlarged at 8-10cm size. Six live babies weighing 1.25, 1.06, 1.15, 1.23, 1.2, and 1.01 Kg were delivered. Neonatologists attended to each of the babies. She required one dose of labetalol 10mg IV as her blood pressure increased to 170/100mmHg. Oxytocin five units IV followed by an infusion of 40 units over four hours was started. The estimated blood loss was 900 ml. A vaginal pack was placed by the surgeon. One unit of packed red cell transfusion was given. The surgery lasted for two hours. She was shifted to the High-Dependency Unit in view of high blood pressure. Thromboprophylaxis was advised to start eight hours after surgery.

She had episodes of high BP in the postoperative ward, which was treated with IV hydralazine 5mg and oral labetalol 200mg twice daily. There was mild bleeding in the postoperative period which was treated with tranexamic acid. Further two units of blood were transfused as blood loss was estimated to be 400 ml in the first 12 hours after cesarean section and Hemoglobin dropped to 8.2g/dL (Table 1). Her blood pressure gradually came down from 156/111 to normal levels in the following days. The hemoglobin level came up to 9.6gm/dL. She had a subsequent uneventful postoperative period and was discharged home on the fourth postoperative day.

## Discussion

The number of multiple pregnancies has increased since the advent of assisted reproduction technologies [1,2]. Antepartum complications including preterm labor, intrauterine growth retardation, gestational diabetes, preeclampsia, and placenta previa are more common in multiple pregnancies [3]. Cesarean section in a mother with a sextuplet pregnancy requires meticulous planning and team effort. An overcrowded operation theatre environment can distract and potentially cause errors in management. PCOS, subfertility, and the consequent need for ARTs in such patients predispose them to maternal and fetal morbidity [4,5]. This makes anesthetic management of cesarean section difficult. Anesthesia and surgery during the ovarian hyperstimulation phase are hazardous when there is associated pleural effusion, ascites, and electrolyte imbalance. Vascular endothelial growth factor and other mediators are thought to cause increased vascular permeability leading to ascites and pleural effusion in OHSS [6]. Hypovolemia, oliguria, electrolyte imbalance (hyponatremia and hyperkalemia), and hemoconcentration can occur. Patients with multiple pregnancies are likely to come for prophylactic cervical cerclage in early pregnancy.

Physiologic changes in pregnancy are exaggerated in multiple pregnancies. Most profound effects occur on the cardiovascular, respiratory, and central nervous systems. This helps to meet the stress and demands of the mother and fetus [7]. There is a relatively greater increase in plasma volume leading to anemia. Heart rate, stroke volume, and cardiac index increase more in multiple pregnancies. Supine hypotension is more severe because of the larger uterus [7]. They may not mount an adequate hemodynamic response to stress because of lower cardiac reserve in multiple pregnancies [8]. Inferior vena cava (IVC) compression leads to epidural venous engorgement, reducing subarachnoid fluid space. This can result in a higher level of spinal anesthesia and subsequent hypotension. There is increased nerve sensitivity to local anesthetics because of increased concentrations of progesterone in multiple pregnancies [9]. Higher progesterone levels in multiple pregnancies stimulate the respiratory center and increase minute ventilation. There is a greater mechanical effect on the diaphragm due to the larger uterus which results in lower functional residual capacity of the lungs [7]. These changes make the mother vulnerable to hypoxia during induction of general anesthesia. The risk of vomiting and pulmonary aspiration is high. The incidence of maternal hemorrhage is high due to uterine atony resulting from an overdistended uterus caused by more fetuses, placental mass, and amniotic fluid. There are more requirements for intravenous fluids, uterotonics, and blood transfusion [2,7].

Regional anesthesia is considered safer than general anesthesia for cesarean sections. Epidural, spinal, and general anesthesia are all acceptable for cesarean section [9]. Our institutional practice is to use spinal anesthesia for emergency cesarean sections. This is because spinal anesthesia is achieved quicker than epidural anesthesia. Intrathecal morphine is used for postoperative analgesia. At least two large-bore intravenous cannulas should be in place and blood should be cross-matched. Aortocaval compression and rapid oxygen desaturation are exaggerated in this population. There is a high chance of postpartum hemorrhage requiring resuscitation and additional uterotonics. The neonatal resuscitation team must be present.

Assisted reproductive technologies and ovulation induction cause several adverse effects on the mother and fetus [10]. This includes multifetal gestations, prematurity, cesarean delivery, placenta previa, abruptio placentae, preeclampsia, and gestational diabetes mellitus [11]. Subfertile females tend to have more pre-existing medical conditions, which increase the risk of maternal morbidity [4,12]. Obstetric hemorrhages have been reported to be increased after assisted reproduction technologies, but the mechanisms involved are unclear [13]. In a retrospective cohort study of over one million US deliveries between 2008 and 2012, severe maternal morbidity was nearly twice as likely in pregnancies conceived with ART compared with non-ART pregnancies [14].

PCOS is frequently associated with fertility problems. She had ovulation induction for her previous two pregnancies also. It is one of the most common endocrinopathies in women of reproductive age, affecting between 5 and 10 percent of women overall [15]. PCOS can be readily diagnosed when women present with the classic features of hirsutism, irregular menstrual cycles, and polycystic ovarian morphology on transvaginal ultrasound. Obesity and sleep apnea are common [15,16]. Women with PCOS show a higher risk of gestational complications, such as GDM, hypertension, and preeclampsia [17,18]. The risk of insulin resistance and GDM is increased in women with PCOS independent of BMI [6]. In a meta-analysis of 27 studies involving 4982 women with PCOS, the odds ratio of developing GDM, pregnancy-induced hypertension, preeclampsia, and preterm birth were 3.43, 3.43, 2.17, and 1.93 respectively, when compared with the general obstetric population [19].

Dyslipidemia is common in PCOS. Women with PCOS have higher LDL-cholesterol and non-HDL-cholesterol, regardless of BMI. This increases cardiovascular risk in pregnancy. The presence of obesity, insulin resistance, hypertension, and impaired glucose tolerance or type 2 diabetes increase the risk of coronary artery disease in women with PCOS [20].

## Conclusions

Anesthetic management of a cesarean section in a sextuplet pregnancy needs to be well-planned. Preanesthetic evaluation should take into account exaggerated changes normally seen during pregnancy. Peripartum hemorrhage should be anticipated. PCOS and ovulation induction add extra risks to the mother and baby. GDM, pregnancy-induced hypertension, and preeclampsia are more common. Ovarian hyperstimulation can occur in early pregnancy after ovulation induction.
